# How does research activity align with research need in chronic subdural haematoma: a gap analysis of systematic reviews with end-user selected knowledge gaps

**DOI:** 10.1007/s00701-023-05618-2

**Published:** 2023-05-30

**Authors:** Conor S. Gillespie, Kwan Wai Fung, Ali M. Alam, Alvaro Yanez Touzet, Jugdeep Dhesi, Ellie Edlmann, Jonathan Coles, David K. Menon, Peter J. Hutchinson, Daniel J. Stubbs, Benjamin M. Davies, On behalf of the ICENI Collaboration

**Affiliations:** 1 Department of Neurosurgery, Department of Clinical Neurosciences, University of Cambridge, CB2 0SZ Cambridge, UK; 2 Department of Neurosurgery, The Walton Centre NHS Foundation Trust, Liverpool, UK; 3 School of Medical Sciences, Faculty of Biology, Medicine and Health, University of Manchester, Manchester, UK; 4 Department of Ageing and Health, Guy’s and St Thomas’ NHS Foundation Trust, London, UK; 5 Division of Anaesthesia, Department of Medicine, University of Cambridge, Cambridge, UK

**Keywords:** Chronic subdural haematoma, Umbrella review, Reporting quality, PRISMA, AMSTAR, ICENI

## Abstract

**Background:**

Chronic subdural haematoma (CSDH) is increasingly common. Although treatment is triaged and provided by neurosurgery, the role of non-operative care, alongside observed peri-operative morbidity and patient complexity, suggests that optimum care requires a multi-disciplinary approach. A UK consortium (Improving Care in Elderly Neurosurgery Initiative [ICENI]) has been formed to develop the first comprehensive clinical practice guideline. This starts by identifying critical questions to ask of the literature. The aim of this review was to consider whether existing systematic reviews had suitably addressed these questions.

**Methods:**

Critical research questions to inform CSDH care were identified using multi-stakeholder workshops, including patient and public representation. A CSDH umbrella review of full-text systematic reviews and meta-analysis was conducted in accordance with the PRISMA statement (CRD42022328562). Four databases were searched from inception up to 30 April 2022. Review quality was assessed using AMSTAR-2 criteria, mapped to critical research questions.

**Results:**

Forty-four critical research questions were identified, across 12 themes. Seventy-three articles were included in the umbrella review, comprising 206,369 patients. Most reviews (86.3%, *n*=63) assessed complications and recurrence after surgery. ICENI themes were not addressed in current literature, and duplication of reviews was common (54.8%, *n*=40). AMSTAR-2 confidence rating was high in 7 (9.6%) reviews, moderate in 8 (11.0%), low in 10 (13.7%) and critically low in 48 (65.8%).

**Conclusions:**

The ICENI themes have yet to be examined in existing secondary CSDH literature, and a series of new reviews is now required to address these questions for a clinical practice guideline. There is a need to broaden and redirect research efforts to meet the organisation of services and clinical needs of individual patients.

**Supplementary Information:**

The online version contains supplementary material available at 10.1007/s00701-023-05618-2.

## Introduction

A chronic subdural haematoma (CSDH) is a collection of aged blood lying in the subdural space [14]. Many CSDH are identified incidentally, but most present with symptoms akin to a slowly evolving stroke [29]. For symptomatic CSDH, surgical evacuation is considered the gold standard of care. CSDHs are associated with age, frailty and co-morbidity and are an increasingly common neurosurgical condition, with operative cases predicted to rise by more than 50% in the next 20 years, as a consequence of ageing populations [20, 30]. In the USA, it is forecast to become the most performed neurosurgical operation by 2030 [2]. CSDHs are associated with a 1-year mortality of up to 32% [18], and significant morbidity either from surgery, or from direct sequalae of disease [18, 28]. The rising incidence among elderly patients, and the associated morbidity and mortality makes CSDH, and its management, an important clinical problem [1, 23].

Targeted clinical research in CSDH, such as investigations into the use of surgical drains [24] and adjuvant steroid therapy [13], has led to a growing evidence base for management of CSDH. However, everyday clinical practice may conflict with this [3]. Moreover, inconsistencies between study findings can leave room for interpretation and barriers to implementation [13, 17, 21]. Further targeted research should only address specific knowledge gaps, and many additional uncertainties that remain within clinical practice may exist [26, 31]. This appears relevant to CSDH, where research has focused on procedural treatment such as surgery within specialist centres, consequently overlooking the burden of disease managed non-operatively, long-term care [9, 27] or challenges that may be encountered by other stakeholders involved in CSDH care, such as Geriatrics, Emergency and Acute Medicine, Anaesthesia and General Practice (Supplementary Figure 1).

These challenges have been recognised by the Improving Care in Elderly Neurosurgery Initiative (ICENI) group, a multi-disciplinary group of experts and stakeholders, who have come together to create comprehensive clinical practice guidelines for CSDH [27]. Clinical practice guidelines are an important tool to translate evidence into practice, enabling multiple sources of evidence to be considered, but also enabling knowledge gaps to be identified, often in the short-term bridged with consensus informed by expert opinion [31]. Aligned with guideline methodology, ICENI has convened a broad multidisciplinary group of professionals, alongside patient and public participants [27]. Working in groups, covering different components of the care pathway, these stakeholders have developed practice-relevant questions to inform targeted systematic reviews and develop clinical practice recommendations.

Recognising that relevant systematic reviews may already exist and to avoid duplicated effort, this guideline process included a framework to use existing reviews, specifically by conducting an ‘umbrella review’ (a systematic review of systematic reviews and/or meta-analyses), identifying those aligned with the guideline questions and using a pre-defined quality threshold for inclusion, including a consideration of their recency.

The primary objective of this article is therefore to report on this gap analysis. However, as the guidelines questions can be considered to represent the needs for clinical practice across CSDH care, and systematic review a surrogate of research activity or direction [5], this analysis can also provide an overview of how ongoing research aligns with current clinical practice need. This is considered and discussed as a secondary objective.

## Methods

### Identification of guideline questions

**Fig. 1:**
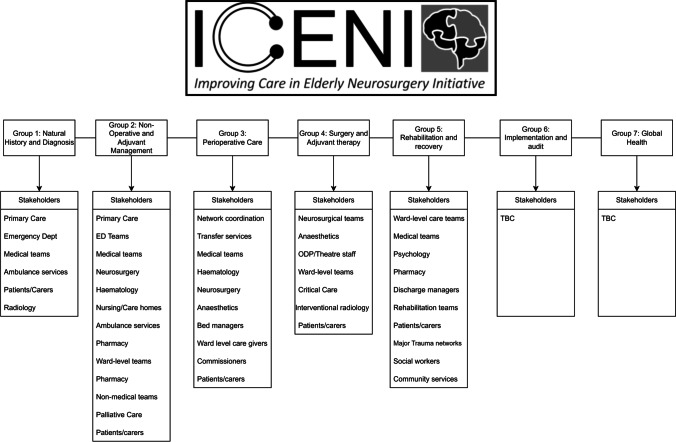
ICENI consensus and stakeholder groups

The ‘Improving Care in Elderly Neurosurgery Initiative’ is a multidisciplinary working group which, with the input and support of professional bodies such as the Society of British Neurological Surgeons (SBNS) and the Neuroanaesthesia and Critical Care Society, is developing a clinical practice guideline for CSDH. The process is supported by further allied organisations including The Neurological Alliance and British Association of Neuroscience Nursing with methodological support from the THiS institute. The group was formed due to a shared consensus that many aspects of CSDH care were unstandardised. Funding was independently secured through application to the National Institute of Academic Anaesthesia with the SBNS identified as the lead sponsor and forward custodian of the final guideline [27]. Building on an initial meeting, working groups were convened to address five areas of CSDH care, i.e. diagnosis, non-operative management, surgical care, peri-operative care and rehabilitation, with work streams around Implementation and Global Health planned at a later stage. For each theme, a dedicated multidisciplinary working group was formed (Fig. 1), and through facilitated discussion, a provisional list of research questions developed. These were initially reviewed using a workshop for patients and members of the public, before final iterations were approved by the steering committee. These research questions thus provide a comprehensive overview of the requirements for the CSDH evidence base.

### Umbrella review

An umbrella review adhering to guidelines outlined by Fusar-Poli and Radua [10] was undertaken to critique the current evidence base. The review was prospectively registered in PROSPERO (CRD42022328562). No changes were made to the original protocol prior to extraction, and anonymised study data are available via the corresponding authors.

#### Search strategy

A comprehensive literature search was performed on 30 April 2022 of 4 databases: Medline, Excerpta Medica Database (Embase), Cochrane Library and the Cumulative Index to Nursing and Allied Health Literature. The exact search strategy for all databases can be found in Supplementary Table 1. We reviewed bibliographies and reference lists of included articles to identify additional studies. Papers were limited to English language due to the feasibility of translation.

#### Inclusion and exclusion criteria

We included all systematic reviews (SRs) published on CSDH, between 1 January 2000 and 30 April 2022. SRs published prior to 2000 were considered almost certainly out of date. An SR was defined by the Cochrane Collaboration as any published, full-text review that attempts to identify, appraise and synthesise available primary research, using prespecified methodology with an aim at minimising bias [6]. Reviews were also included if they contained the phrase ‘systematic review’ in the title or abstract, or if papers described their search strategy as ‘systematic’ or ‘comprehensive’. Studies were excluded if they were conference abstracts, were correspondence, assessed acute subdural haematoma (ASDH) only, assessed both ASDH and CSDH (and it was not possible to distinguish the two), were literature reviews and were invited editorial reviews. Inclusion was determined by two authors (CSG, KWF), with conflict settled by mutual agreement or involvement of a third author (BMD).

#### Data extraction

Independent data extraction was performed in duplicate, by two authors (CSG, KWF) using a standardised pre-piloted data collection proforma (Supplementary Table 2). The following variables were extracted: year of publication, country, continent, journal published; type of SR (SR, meta-analysis, umbrella review); whether the review described adhering to the Preferred Reporting in Systematic Reviews and Meta-Analysis (PRISMA) guidelines or a similar review had been published prior to its publication; number of studies and participants; review typology; domains; and classification. Reporting quality was assessed by calculating the PRISMA checklist adherence [22], and overall quality of review results calculated according to the Assessing the Methodological Quality of Systematic Reviews-2 tool (AMSTAR-2). An AMSTAR-2 overall interpretation score was calculated (‘high’, ‘moderate’, ‘low’ or ‘critically low’) [25]. Formal risk of bias assessment was not performed.

### Definitions

In our review, country was defined as the country of first listed affiliation of the first author. Review typology was defined according to Munn et al. [19], and review domains defined a priori according to the ICENI themes, and separately, a previously defined thematic analysis of CSDH education resources (Gillespie et al, unpublished data, 2022). Manuscripts were considered ‘similar’ if they reported the same research theme as an article published previously (i.e. recurrence rates following middle meningeal artery [MMA] embolization). If the article assessed the same research theme, was not published as part of a ‘review update’ by the same group of authors and was included the same outcomes, it was considered ‘duplicate’.

#### Analysis

Critical research questions ratified by the ICENI steering group were first grouped into topics by two authors (BMD and DJS). These were then matched to the review topics, with activity or lack of activity summarised using descriptive statistics.

The quality of identified reviews according to AMSTAR-2 was summarised using descriptive statistics, with results for individual components presented separately. Normally distributed variables were summarised with mean and standard deviation (SD), and non-parametric variables as medians and inter-quartile ranges (IQRs). All analysis and graphical representation was performed using R v4.0.2 (ggplot and tidyverse packages).

## Results

### Critical research questions

Forty-four questions were generated from the ICENI working groups and ratified by the steering committee (Table 1). These were felt to represent 12 distinct themes: anticoagulation (6 questions), communication (6 questions), decision making (2 questions), anaesthesia and surgical scheduling (7 questions), transfer and pathway (7 questions), perioperative care (3 questions), palliative care (3 questions), postop and recovery (5 questions), natural history (2 questions), surgical technique (3 questions) and MMA embolization (3 questions).

**Table 1:** ICENI research questions, and themes

Theme	*Q*	Research questions
Anticoagulant	13	In patients with cSDH who are not undergoing surgery (P) do antithrombotic drugs (e.g. anticoagulants, antiplatelets) (I) increase the risk of disease related complications (e.g. expansion (O) compared to those who do not take such agents (C)
	14	In patients with cSDH who are not undergoing surgery (P) does discontinuation of antithrombotic agents (I) improve disease and safety related outcomes (O) compared to continuing these agents (C)
	15	In patients with cSDH (P) do antithrombotic drugs (e.g., anticoagulants, antiplatelet agents) (I) increase the risk of treatment related complications (O) compared to those who are not taking such drugs(C)?
	16	Does early (I) vs late (C) recommencement of anticoagulation increase the risk of recurrence or other complications (O) in patients recovering from cSDH surgery?
	17	In patients with cSDH scheduled for surgery who are taking an antithrombotic medication (P) what is the impact of using pharmacological or other (e.g. platelet) reversal (I) on perioperative outcomes (O) compared to standard care (C)
	18	In patients with cSDH who have undergone surgery (P) what is the impact of early (<72 hrs) (I) commencement of prophylactic LMWH on perioperative thromboembolic and rebleeding (incl recollection) (O) compared to standard care (C)
Communication and decision-making	1	In patients with a radiological finding of a cSDH (P) does the use of standardised tools for neurosurgical referral and intervention (I) improve patient, system, and clinical outcomes (O) compared to standard care (C)
	2	In patients with a symptomatic, cSDH (P) does active neurosurgical management (including surgery, MME, or adjuvant medical therapies) (I) compared to conservative or medical management (C) improve patient, system, clinical outcomes (O)?
	3	In patients with an incidental cSDH (P) does active neurosurgical management (including surgery, MME or adjuvant medical therapies) (I) compared to conservative or medical management (C) improve patient, system, clinical outcomes (O)?
	6	In patients with a cSDH being discussed with a neurosurgeon (P), do standardised communication tools (e.g. structured referral proformas or decision making tools) (I) improve surgical decision making (O) compared to standard care (C)?
	7	In patients with cSDH being triaged for surgery (P), does the explicit identification and consideration of patient and family recovery priorities (I), improve patient, provider, and clinical outcomes (O),
	8	In patients with cSDH being triaged for surgery (P) does a patient and family discussion around perioperative risks and benefits led by a specialist (e.g. neurosurgeon) (I) improve patient, provider, and clinical outcomes (O) compared to a non-specialist led discussion? (C)
Anaesthesia and surgical scheduling	22	In patients undergoing surgery for cSDH (P) does the use of local anaesthesia (I) versus general anaesthesia (C) improve patient, system, and clinical outcomes (O)
	23	In patients having surgery for cSDH (P) does protocolised or strict blood pressure control (e.g. avoidance of hypotension) (I) improve postoperative outcomes (O) compared to routine management (C)
	24	In patients having surgery for cSDH (P) does advanced or invasive monitoring (I) improve perioperative blood pressure control (O) compared to routine monitoring (C)
	25	In patients with a cSDH scheduled for surgery (P) does early surgery (I) improve patient, system, and clinical outcomes (O) compared to routine management (C)
	26	Do patients with a cSDH scheduled for surgery (P) who face a cancellation / delay / prolonged fasting (I) compared to those who do not (C) haved improved patient, system, and clinical outcomes (O)
	27	In patients with a cSDH scheduled for surgery (P) does in-hours surgery (I) improve patient, system, and clinical outcomes (O) compared to out-of hours surgery (C)
	41	In patients undergoing a procedural intervention for chronic subdural haematoma (P) does provision of surgical/procedural/anaesthetic care by a ‘senior’ (I) (i.e. consultant level) provider vs ‘junior’ (i.e. non-consultant level) (C) affect patient, system, and provider outcomes (O)
Transfer and pathway	9	In patients with cSDH being transferred for surgery (P) how does an optimized/protocolized transfer (I) compared to routine care (C) affect patient, system, clinical outcomes (O)
	10	In patients with cSDH being transferred for surgery (P) does immediate transfer to tertiary centre (I) compared to routine care (C) improve patient, system, clinical outcomes (O)
	11	For cSDH patients (P), what is the role of technology (I) (e.g., QR codes, e-communication) compared with standard care (C) in facilitating communication between centres (incl. transfer of relevant patient information)((O
	12	In patients with CSDH transferred for surgery (P) does repeating blood tests (I) compared to using those communicated from original hospital (C) improve patient, system, clinical outcomes (O)
	30	In patients presenting to healthcare services with an undiagnosed cSDH (P) do standardised symptom checklists (I) improve time to diagnosis and treatment decision (O) compared to routine care? (C)
	33	In patients who have undergone interventional treatment for a cSDH and being discharged or transferred to another centre (P), do standardised communication tools (e.g. structured proformas) (I) improve patient, system, and clinical (O) compared to standard care (C)?
	4	In patients with a CSDH (both operative and non-operative) is their outcome (both patient and clinical) (O) improved if they receive ongoing care (e.g. rehabilitation, medical management) in a specialist (I) (neurosciences or rehabilitation facility) compared to non-specialist (secondary care) setting?
Perioperative care	19	Does the use of objective assessment tools (e.g. such as those used in Comprehensive Geriatric Assessment: frailty, cognition, multi-morbidity) to identify and optimise high-risk patients (I) in patients presenting with a cSDH (P) improve patient, system, and clinical outcomes (O) compared to standard care (C) ?
	20	In patients with a cSDH (P) Does protocolised multidisciplinary care (e.g. co-management with a geriatrician) (I) improve patient, system, and clincial outcomes (O) compared to standard care (C)?
	21	Does assessing and optimising delirium risk (I) in cSDH patients who are scheduled for surgery (P) help to prevent, diagnose and treat this condition (O) compared to standard care? (C)
Palliative Care	36	In patients with a symptomatic cSDH suspected not to benefit from treatment (P) does assessment by a nominated specialist (e.g. neurosurgeon) (I) improve diagnostic accuracy, patient, and family relevant outcomes (O) compared to standard care?
	37	Is delivery of palliative care by specialists (e.g. specialist doctor or nurse) (I) associated with improved patient and family outcomes (O) for individuals with cSDH in whom this is felt to be an end-of-life diagnosis (P) compared to non-specialist delivered care (C)?
Postop and recovery	28	Does standardised postoperative posture support and mobilisation rules (e.g. routine use of a supine position) (I) improve patient, system, and clinical outcomes (O) after cSDH surgery(P) compared to routine care (C) ?
	31	In patients with a cSDH (both operatively and conservatively managed) (P) does the use of standardised tools to assess ongoing rehabilitation requirements (I) improve patient, system, and clinical outcomes (O) compared to standard care?
	32	In patients who have had interventional treatment for cSDH (P) does protocolised post-operative care and standardised discharge criteria (I) improve patient, system (e.g. time to discharge, DToC rates), and clinical outcomes (O) compared to standard care?
	34	In patients who have had surgery for cSDH (P) does the provision of standardised ‘red-flag’ checklists (I) improve time-to-diagnosis of symptomatic recurrence(O) compared to standard care (C)
	4	In patients with a CSDH (both operative and non-operative) is their outcome (both patient and clinical) (O) improved if they receive ongoing care (e.g. rehabilitation, medical management) in a specialist (I) (neurosciences or rehabilitation facility) compared to non-specialist (secondary care) setting?
Natural history	29	What factors (I) are most associated with an increased risk for developing cSDH (O) among older adults in the community (P) compared with older adults without these factors (C)?
	35	In patients with a CSDH triaged for non-operative management (P), does active surveillance (e.g. interval CT imaging) (I) compared to expectant management (C) improve patient, system, and clinical outcomes (O)
Surgical technique	38	In patients who undergo surgical treatment for cSDH (P) does craniotomy (I) improve patient, system (e.g. time to discharge, DToC rates), and clinical outcomes (O) compared to burr holes?
	40	In patients who undergo surgical treatment for cSDH (P) does (Drain Variation X) (I) improve patient, system (e.g. time to discharge, DToC rates), and clinical outcomes (O) compared to subdural catheter on free drainage?
	41	In patients undergoing a procedural intervention for chronic subdural haematoma (P) does provision of surgical/procedural/anaesthetic care by a ‘senior’ (I) (i.e. consultant level) provider vs ‘junior’ (i.e. non-consultant level) (C) affect patient, system, and provider outcomes (O)
MMA embolisation	42	In patients with an incidental cSDH (P) does MMA (I) improve patient, system (e.g. time to discharge, DToC rates), and clinical outcomes (O) compared to surveillance and risk factor modification alone?
	43	In patients with a symptomatic cSDH (P) does MMA (I) improve patient, system (e.g. time to discharge, DToC rates), and clinical outcomes (O) compared to surgical evacuation?
	44	In patients with a symptomatic cSDH (P) does MMA in addition to surgery (I) improve patient, system (e.g. time to discharge, DToC rates), and clinical outcomes (in particular recurrence) (O) compared to surgical evacuation?

### Umbrella review

Seventy-three articles (Supplementary Table 3) reporting on 1914 studies and 206,379 patients were included (Supplementary Figure 2). A list of full-text articles screened, but excluded from the review, is included in Supplementary Table 4.

Study characteristics are outlined in Table 2. Almost all papers (98.6%, *n*=72/73) were published between 2010 and 2022. Most articles were from China (32.9%, *n*=24), followed by USA (20.5%, *n*=15), UK (8.2%, *n*=6), Netherlands (8.2%, *n*=6) and Canada (8.2%, *n*=6). The most common publishing journals were World Neurosurgery (21.9%, *n*=16), Acta Neurochirurgica (9.6%, *n*=7) and Frontiers in Neurology (6.8%, *n*=5). The median number of studies included per review was 13 (IQR 7–21), and the median number of participants was 1119 (IQR 441–3149). 76.7% (*n*=56) explicitly referenced following PRISMA guidelines. The most common review typology was effectiveness (71.2%, *n*=52). The overall mean adherence to PRISMA guidelines was 58.5% (SD 16.9).

**Table 2:** Study characteristics

Baseline characteristics	Value (%) [SD] (IQR)
Total studies included	73
Review types	Frequency
Systematic review and meta-analysis	50 (68.5)
Systematic review	22 (30.1)
Umbrella review	1 (1.4)
Continent of authors	Frequency
Asia	29 (39.7)
North America	21 (28.8)
Europe	19 (25.8)
Other	4 (5.7)
Journal	Frequency
World Neurosurgery	16 (21.9)
Acta Neurochirurgica	7 (9.6)
Frontiers in Neurology	5 (6.8)
Neurosurgical Review	3 (4.1)
Journal of Neurotrauma	3 (4.1)
Medicine (Baltimore)	3 (2.7)
Journal of Neurosurgery	2 (2.7)
Journal of Clinical Neurosciences	2 (2.7)
Journal of Neurointerventional Surgery	2 (2.7)
British Journal of Neurosurgery	2 (2.7)
Other	28 (38.4)
Study details	Frequency
Number of included studies	13 (7–21)
Number of included participants	1119 [441–3149]
Total number of included participants	206,379
Review typology	Frequency
Effectiveness	52 (71.2)
Prevalence and/or incidence	3 (4.1)
Methodological	3 (4.1)
Other	15 (20.6)
Mentioned reporting according to PRISMA	Frequency
Yes	56 (76.7)
No	17 (23.3)

*SD* standard deviation, *IQR* inter-quartile range

#### Content and domain reporting

**Fig. 2:**
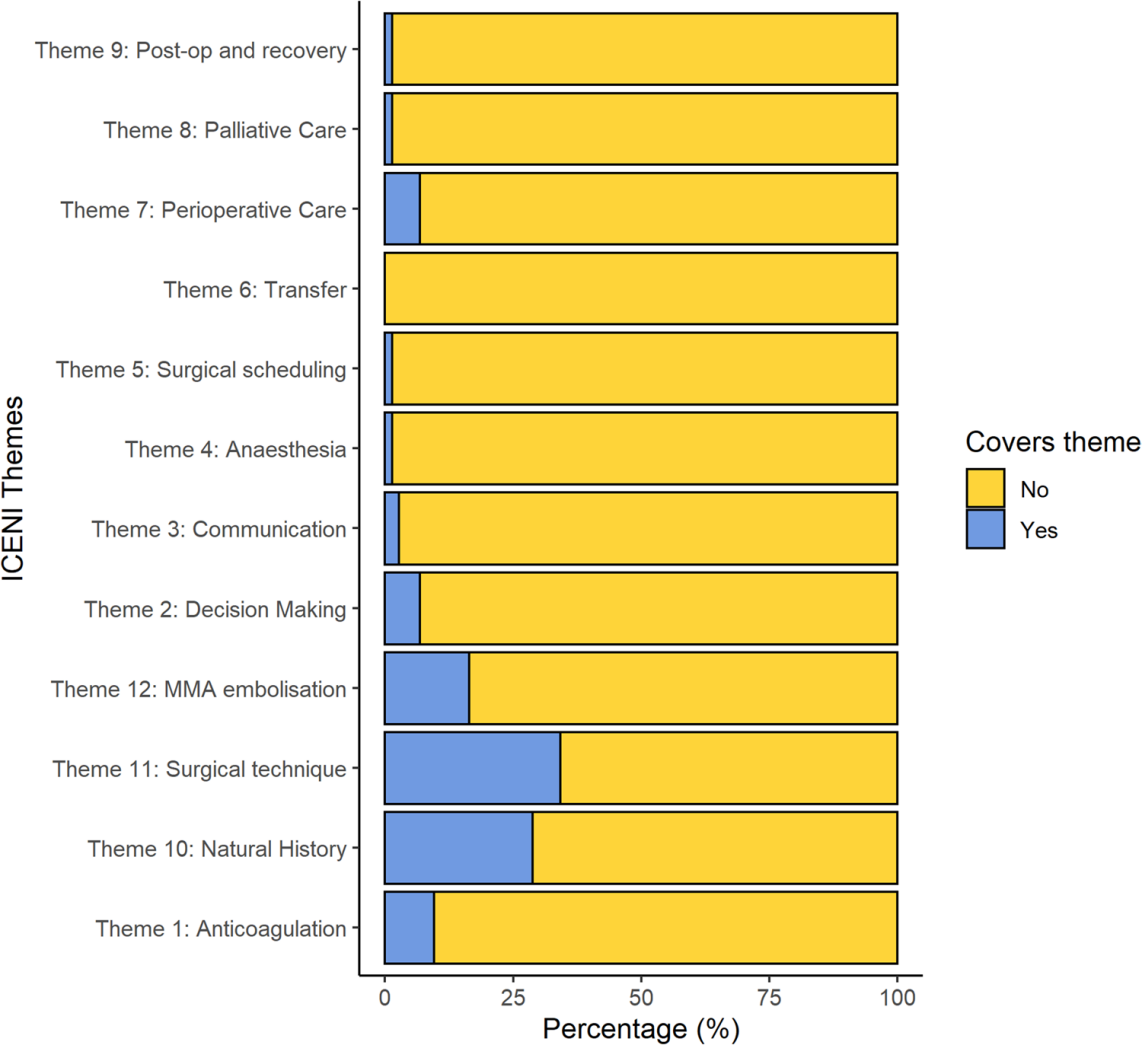
Bar chart of ICENI themes included in existing CSDH reviews

The number of reviews examining each ICENI theme is shown in Table 3, and Fig. 2. The three most common themes addressed were surgical technique (34.2%, *n*=25), natural history (28.8%, *n*=21) and MMA embolisation (16.4%, *n*=12). Content was additionally categorised according CSDH themes identified through thematic analysis of CSDH education resources. These are shown in Supplementary Table 5. In total, 86.3% (*n*=63) of reviews assessed complications and recurrence, 65.8% (*n*=48) survival and performance outcomes, 50.7% (*n*=37) non-surgical management and 43.8% (*n*=32) surgical management. No reviews assessed quality of life.

**Table 3:** ICENI themes, number of research questions and number of reviews for each category

Theme	Number of research questions (*N*)	Reviews identified (%)
Anticoagulant	6	7 (9.6)
Decision-making	6	5 (6.8)
Communication	2	2 (2.7)
Anaesthesia and surgical scheduling	7	1 (1.4)
Transfer	7	0 (0.0)
Perioperative care	3	5 (6.8)
Palliative care	3	0 (0.0)
Postop and recovery	5	1 (1.4)
Natural history	2	21 (28.8)
Surgical technique	3	25 (34.2)
MMA embolisation	3	12 (16.4)

In total, 55 studies were identified as work similar to that of previously published reviews (Supplementary Table 6). The number of ‘duplicate’ manuscripts was 54.8% (*n*=40). The most duplicated (‘similar’ or ‘very-similar’) reviews related to MMA embolisation (*n*=9), corticosteroid use (*n*=7) and drain use (*n*=6).

#### AMSTAR-2 reporting quality

The quality scores for each component are shown in Table 4. The highest reported field was item 8 (‘including details of studies’ (94.5%, *n*=69/73)) and item 10 (‘declaring review author funding’ (90.4%, *n*=66)). The lowest reported fields were item 10 (‘assessing funding of included papers in review’ (27.4%, *n*=20)), and item 2, (‘referring to a study protocol’ (32.9%, *n*=24)). Overall, 7 reviews (9.6%) were classified as having high confidence in the results of the review. A further 8 studies (11.0%) were classified as having moderate confidence in results, 10 (13.7%) as low confidence and 48 (65.7%) critically low confidence.

**Table 4:** AMSTAR-2 reporting of CSDH reviews

Item number	Description	Frequency (%)
1	PICO description	62 (84.9)
2	Review methods and protocol deviations	24 (32.9)
3	Explain study designs for inclusion	56 (76.7)
4	Comprehensive search strategy	38 (52.1)
5	Duplicate study selection	55 (75.3)
6	Duplicate data extraction	55 (75.3)
7	List of excluded studies with justification	29 (39.7)
8	Description of included studies	69 (94.5)
9	Risk of bias for included studies	45 (61.6)
10	Funding sources for included studies	20 (27.4)
11	Meta-analysis statistics	24 (32.9)
12	Meta-analysis risk of bias	21 (42.9)
13	Risk of bias in discussion	44 (60.3)
14	Explanation of heterogeneity	48 (65.8)
15	Publication bias	29 (59.2)
16	Conflict of interest reporting	66 (90.4)
Confidence rating		
1	High	7 (9.6)
2	Moderate	8 (11.0)
3	Low	10 (13.7)
4	Critically low	48 (65.8)

*Number lower as only applies to systematic reviews with meta-analysis

### Gap analysis of ICENI themes matched to AMSTAR-2 score

**Fig. 3:**
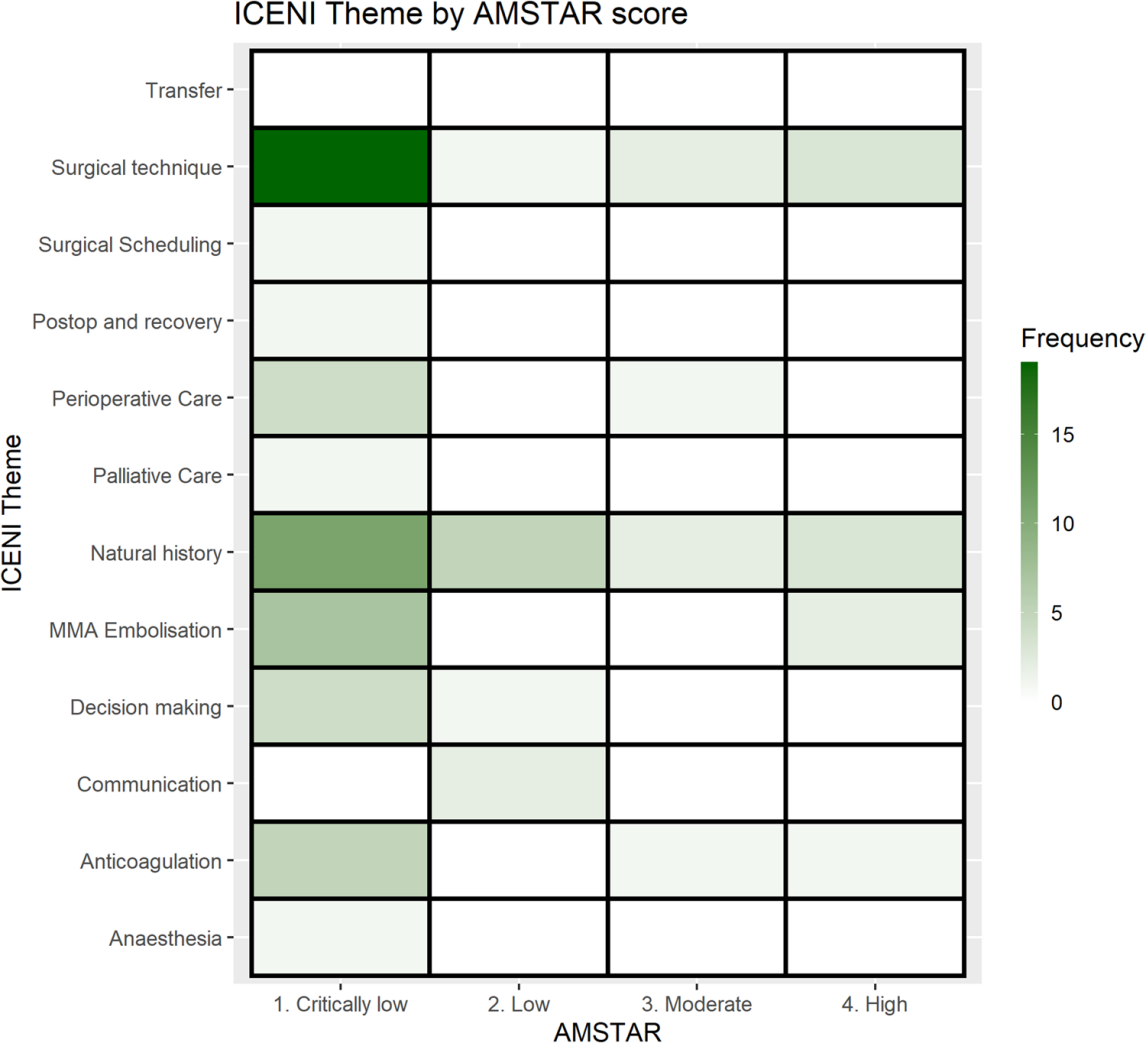
Heatmap gap analysis of ICENI themes, stratified by AMSTAR-2 confidence rating (white indicates zero reviews available

The gap analysis of the 12 ICENI themes, mapped to AMSTAR-2 score, is shown in Fig. 3. In total, there was one high confidence review on anticoagulation, three on natural history, two on surgical technique and one on MMA embolisation. There was one moderate confidence review on perioperative care, two on natural history, two on surgical technique and two on MMA embolisation. All other reviews scored either low or critically low in confidence level when addressing ICENI themes. The reviews with high AMSTAR ratings are shown below in Online Supplementary Table 7.

## Discussion

### Summary of findings

This umbrella review and gap analysis identified that available systematic reviews on CSDH have critically low confidence in quality assessment. The CSDH evidence base focusses almost exclusively on procedural interventions, and binary outcomes (such as recurrence and mortality). Only 7 reviews targeted, with sufficient quality, questions posed for the proposed ICENI CSDH guidelines—indicating many areas relevant to CSDH management that are not addressed by current systematic reviews.

However, even within this narrow focus of procedural interventions, there exists a duplication of effort, and very often reviews were poorly placed to inform care; for example, almost two-thirds of reviews were categorised as having critically low confidence in their results according to AMSTAR-2. It is important to note that each component of AMSTAR-2 is not considered by all to have equal weighting or significance, for example factors such as protocol registration and the funding of included studies, which were the most poorly reported domains [12, 25]. Further, a risk of bias assessment was not formally completed. However, corroborating this finding, PRISMA compliance was also low.

Systematic reviews are important and influential—they inform future research, and are often required by research funders for trial applications [5, 11, 26]. Consequently, albeit a surrogate, the focus and activity within an umbrella review is an indication of the broader research environment. That CSDH is therefore focused on procedural interventions and their consequences is unsurprising. At present, despite a variety of other stakeholders that may be involved [29], the common focal point for anyone diagnosed with a CSDH is a consultation with Neurosurgery to determine whether treatment is required. The majority of CSDH research is undertaken by neurosurgical teams, and this therefore reflects their principal requirements [9]. Thus, whilst the evidence base around surgical management has improved and is ready to inform care, broader challenges remain. This reinforces the importance of the ICENI initiative, and specifically the role of multi stakeholder engagement to identify unsolved yet important challenges in the clinical pathway [4, 8, 9, 27].

**Fig. 4:**
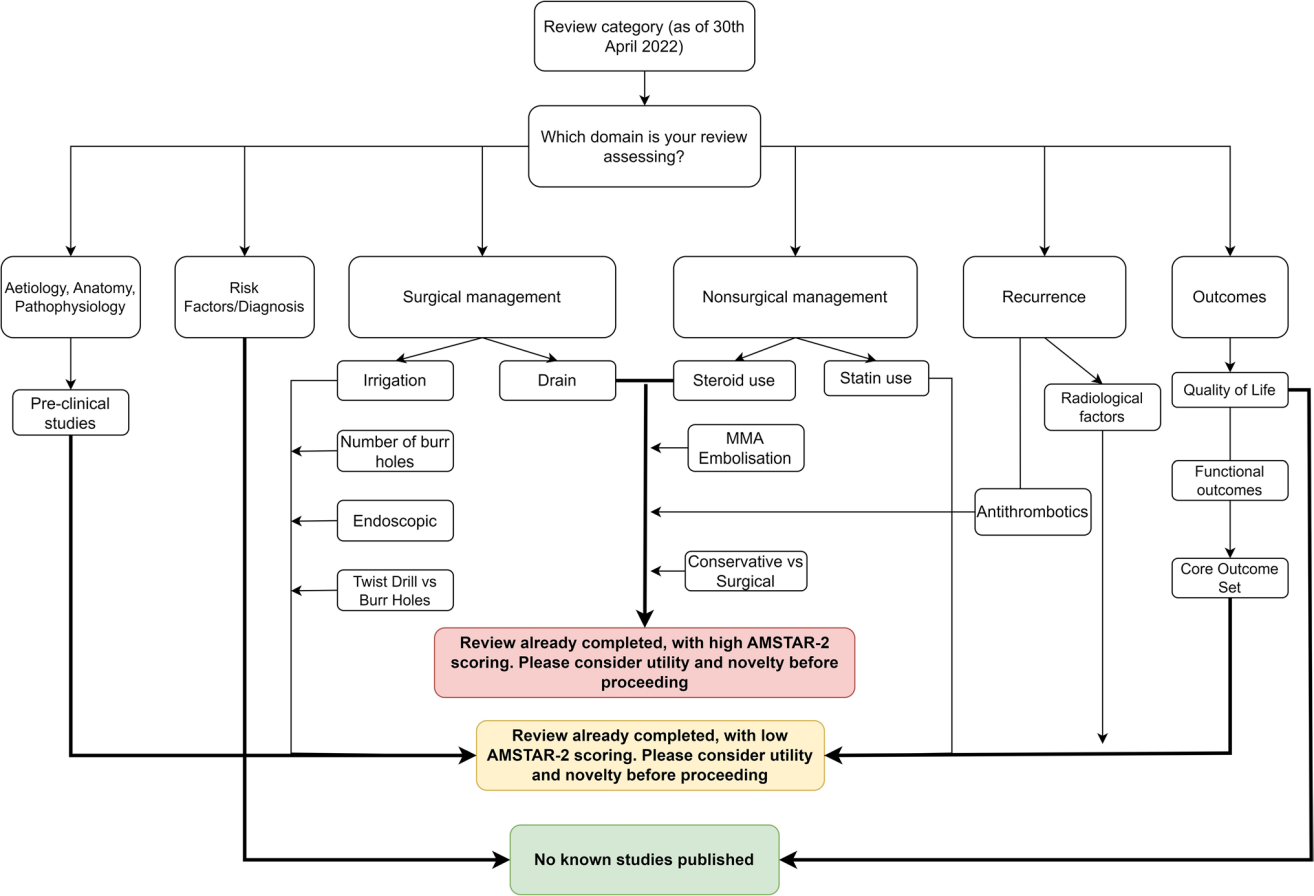
Decision aid to guide future systematic reviews on Chronic Subdural Haematoma

This study therefore has simple but important implications for improving care in CSDH. Firstly, the information gathered by this manuscript and literature gaps identified can be used to guide future reviews [9]. In particular, peri-operative anaesthesia [16] and quality of life appear areas of unmet need. Second, we have produced a decision flow-chart (Fig. 4) that can be used to guide the process of selection of systematic reviews and meta-analyses, to avoid review saturation as a consequence of duplication of work produced in the CSDH field in the last 5 years. Further, it reinforces the importance of aiming for quality when conducting reviews, to ensure their conduct can inform care, for example through uptake in clinical practice guidelines. Broader strategies including PROSPERO registration, and PRISMA Reporting Guidelines have been important interventions, and adherence is key to ensuring optimal reviews are performed. Finally, it reinforces the need to capture a multi-stakeholder perspective when seeking to solve a clinical problem. Research shows that professionals gravitate towards procedural research, in contrast to priorities set by organisations such as the James Lind Alliance [7, 15].

## Conclusion

Systematic reviews and meta-analysis on management of CSDH focus on procedural interventions, such as surgery or MMA embolisation. Further, they are poorly compliant with recommended reporting checklists and are often of low quality. Many themes identified as critical to inform clinical care by multidisciplinary groups remain to be explored in CSDH. New evidence synthesis that adheres to available checklists, and addresses these gaps, is therefore required to strengthen the current limited evidence base, avoid bias and enhance CSDH care.

## Supplementary information

ESM 1 (DOCX 827 kb)

ESM 2 (DOCX 48 kb)
