# Supplementary material for: How does research activity align with research need in chronic subdural haematoma: a gap analysis of systematic reviews with end-user selected knowledge gaps
Source: Acta Neurochir (Wien). 2023 May 30;165(7):1975–86. doi: 10.1007/s00701-023-05618-2 (PMC10319658; doi:10.1007/s00701-023-05618-2)
Supplement: Supplementary file 1 — (DOCX 827 kb) [file 701_2023_5618_MOESM1_ESM.docx]

**Supplementary Figures**

**Supplementary Figure 1: Flow diagram of possible treatment pathway in CSDH, and multi-disciplinary teams that may be involved at each stage.**

**
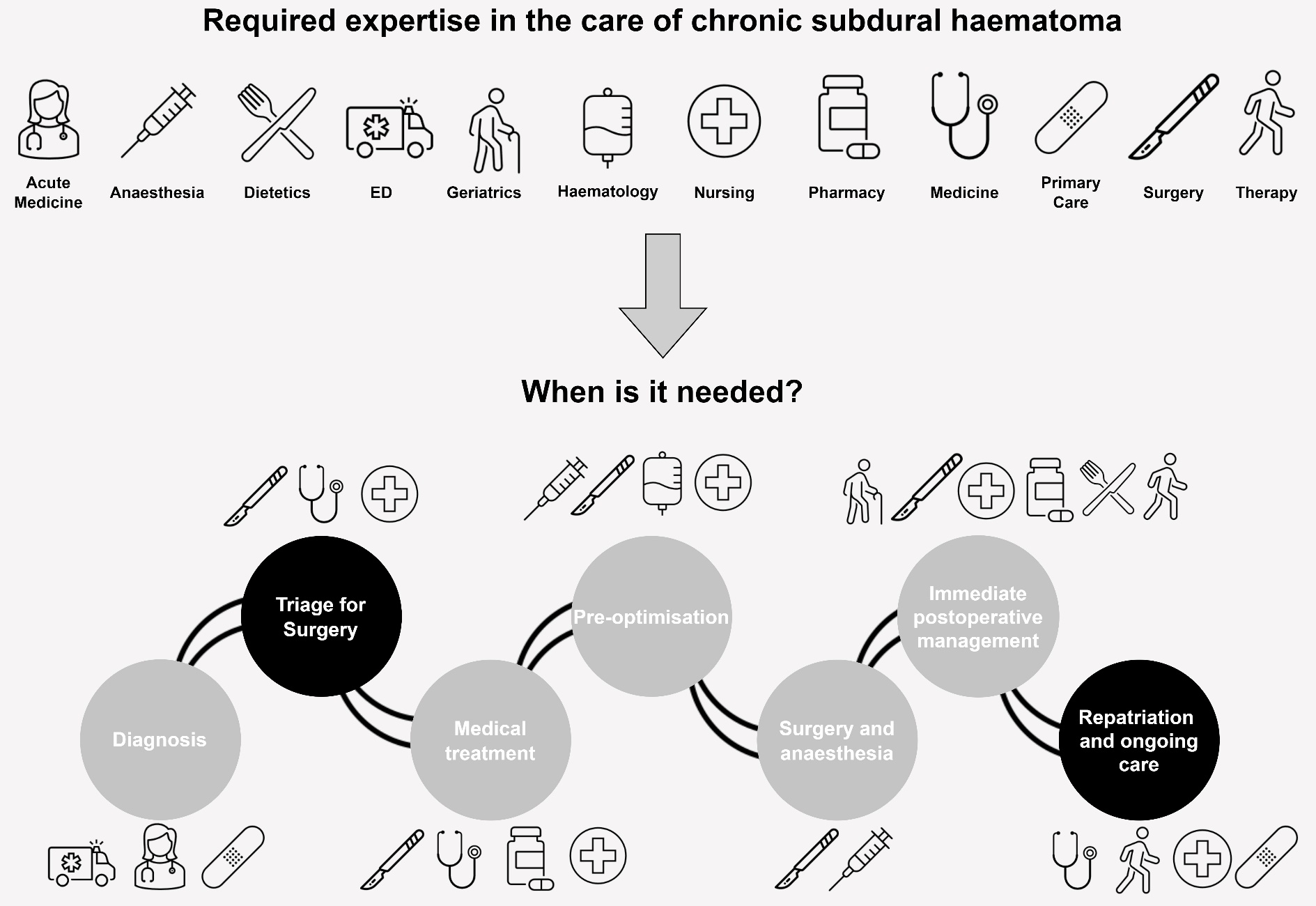
**

**Supplementary Figure 2: PRISMA flow chart**

**
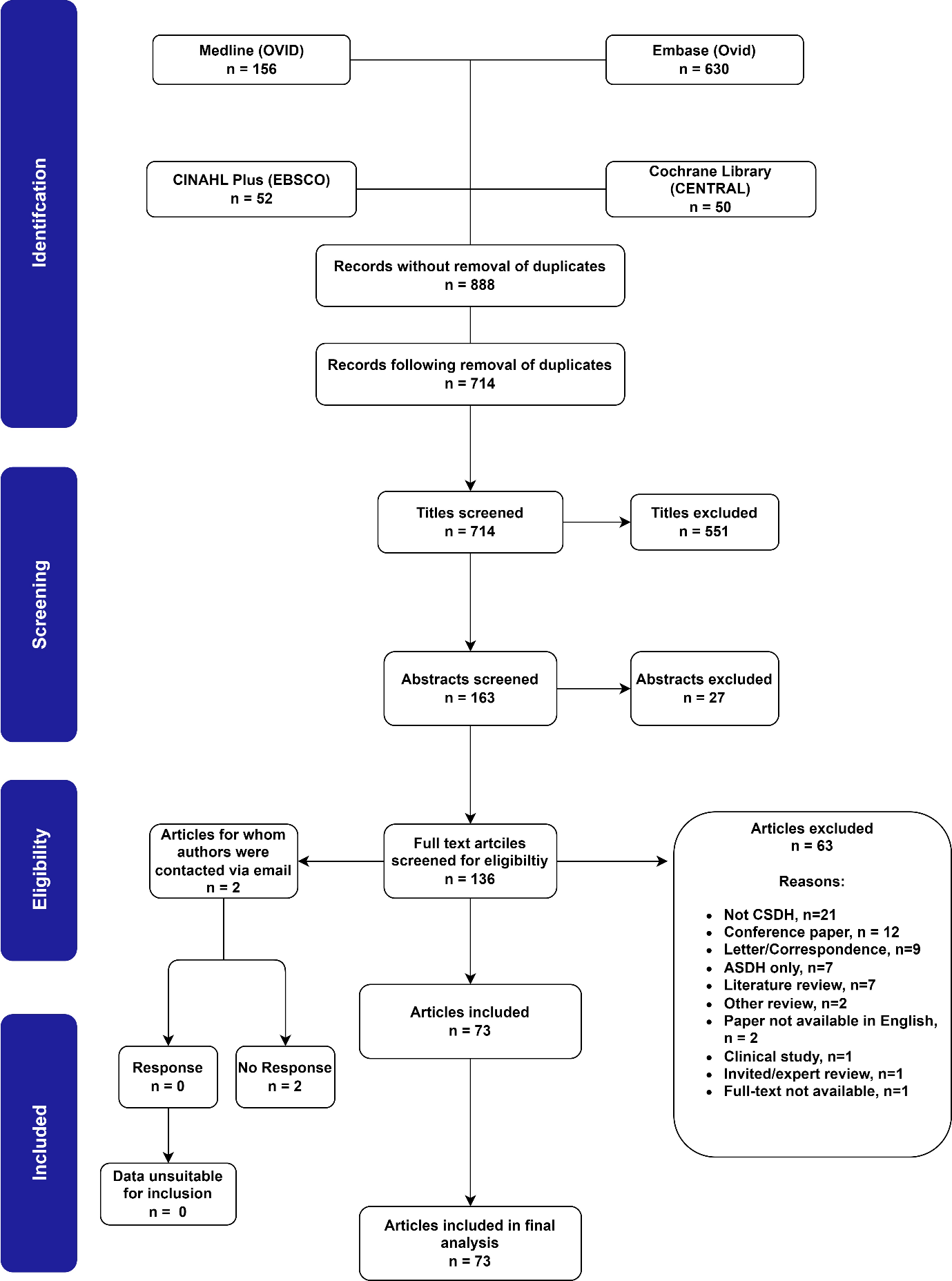
**
