# Supplementary material for: How does research activity align with research need in chronic subdural haematoma: a gap analysis of systematic reviews with end-user selected knowledge gaps
Source: Acta Neurochir (Wien). 2023 May 30;165(7):1975–86. doi: 10.1007/s00701-023-05618-2 (PMC10319658; doi:10.1007/s00701-023-05618-2)
Supplement: Supplementary file 2 — (DOCX 48 kb) [file 701_2023_5618_MOESM2_ESM.docx]

**Online Supplementary tables**

Online Supplementary Table 1- Medline (Ovid), Embase (Ovid), CINAHL Plus (EBSCO) and Cochrane Library (CENTRAL) search strategies (last conducted 30^th^ April 2022).

**MEDLINE**

| Search | Query | Results (30/04/22) |
| --- | --- | --- |
| 1 | Chronic Subdural hematoma/ | 1635 |
| 2 | ((chronic or non-traumatic or nontraumatic or spontaneous*) and (subdural or "sub dural") and (haematoma* or hematoma* or haemorrhag* or hemorrhag* or bleed*)).mp | 5384 |
| 3 | 1 or 2 | 5384 |
| 4 | Systematic reviews system as topic[mh] | 194702 |
| 5 | Systematic review*.mp | 255514 |
| 6 | Meta-analysis[mh] | 242824 |
| 7 | Meta-analysis as topic | 159501 |
| 8 | Meta-analy* | 266138 |
| 11 | Evidence review* | 3684 |
| 12 | Review of evidence | 19499 |
| 13 | Review of the evidence | 38390 |
| 14 | Evidence overview* | 57 |
| 15 | Overview of evidence | 395 |
| 16 | Overview of the evidence | 1526 |
| 17 | Evidence synthesis | 6103 |
| 18 | Synthesis of evidence | 1140 |
| 19 | Synthesis of the evidence | 2412 |
| 20 | reference list* | 20742 |
| 21 | bibliograph* | 45201 |
| 22 | hand search* | 7958 |
| 23 | manual search* | 5410 |
| 24 | relevant journals | 1287 |
| 25 | search strategy OR search criteria OR systematic search OR study selection OR data extraction | 71784 |
| 26 | search the literature OR searched the literature OR literature search OR literature searched OR literature was searched | 62396 |
| 27 | medline OR pubmed OR cochrane OR embase OR psychlit OR psyclit OR psychinfo OR psycinfo OR CINAHL OR science citation index | 315108 |
| 28 | 4 OR 5 OR 6 OR 7 OR 8 OR 9 OR 10 OR 11 OR 12 OR 13 OR 14 OR 15 OR 16 OR 17 OR 18 OR 19 OR 20 OR 21 OR 22 OR 23 OR 24 OR 25 | 600877 |
| 29 | 3 AND 26 | 156 |
| **CDSR/COCHRANE LIBRARY** | | |
| Search | Query | Results |
| 1 | ((chronic or non-traumatic or nontraumatic or spontaneous*) and (subdural or "sub dural") and (haematoma* or hematoma* or haemorrhag* or hemorrhag* or bleed*)).mp | 392 |
| 2 | Cochrane reviews or protocols only | 50 |
| **CINAHL PLUS** | | |
| Search | Query | Results (30/04/22) |
| 1 | MH Chronic Subdural | 466 |
| 2 | ((chronic or non-traumatic or nontraumatic or spontaneous*) and (subdural or "sub dural") and (haematoma* or hematoma* or haemorrhag* or hemorrhag* or bleed*)) | 633 |
| 3 | S1 OR S2 | 5673 |
| 4 | Systematic reviews system as topic[mh] | 181474 |
| 5 | Systematic review*.mp | 182910 |
| 6 | Meta-analysis[mh] | 104458 |
| 7 | Meta-analysis as topic | 104458 |
| 8 | Meta-analy* | 105610 |
| 11 | Evidence review* | 169295 |
| 12 | Review of evidence | 154803 |
| 13 | Review of the evidence | 154803 |
| 14 | Evidence overview* | 9246 |
| 15 | Overview of evidence | 9221 |
| 16 | Overview of the evidence | 9221 |
| 17 | Evidence synthesis | 14154 |
| 18 | Synthesis of evidence | 14154 |
| 19 | Synthesis of the evidence | 14154 |
| 20 | reference list* | 12333 |
| 21 | bibliograph* | 12948 |
| 22 | hand search* | 6700 |
| 23 | manual search* | 5410 |
| 24 | relevant journals | 4801 |
| 25 | search strategy OR search criteria OR systematic search OR study selection OR data extraction | 138105 |
| 26 | search the literature OR searched the literature OR literature search OR literature searched OR literature was searched | 66093 |
| 27 | medline OR pubmed OR cochrane OR embase OR psychlit OR psyclit OR psychinfo OR psycinfo OR CINAHL OR science citation index | 164949 |
| 28 | S4 OR S5 OR S6 OR S7 OR S8 OR S9 OR S10 OR S11 OR S12 OR S13 OR S14 OR S15 OR S16 OR S17 OR S18 OR S19 OR S20 OR S21 OR S22 OR S23 OR S24 OR S25 | 429908 |
| 29 | S3 AND S26 | 52 |
| **EMBASE** | | |

| Search | Query | Results (30/04/22) |
| --- | --- | --- |
| 1 | Subdural hematoma/ | 19213 |
| 2 | ((chronic or non-traumatic or nontraumatic or spontaneous*) and (subdural or "sub dural") and (haematoma* or hematoma* or haemorrhag* or hemorrhag* or bleed*)).mp | 6993 |
| 3 | 1 or 2 | 20055 |
| 4 | Systematic reviews system as topic[mh] | 342632 |
| 5 | Systematic review*.mp | 446207 |
| 6 | Meta-analysis[mh] | 244461 |
| 7 | Meta-analysis as topic | 357685 |
| 8 | Meta-analy* | 381818 |
| 11 | Evidence review* | 4127 |
| 12 | Review of evidence | 21631 |
| 13 | Review of the evidence | 42613 |
| 14 | Evidence overview* | 48 |
| 15 | Overview of evidence | 453 |
| 16 | Overview of the evidence | 1737 |
| 17 | Evidence synthesis | 6711 |
| 18 | Synthesis of evidence | 1223 |
| 19 | Synthesis of the evidence | 2636 |
| 20 | reference list* | 24363 |
| 21 | bibliograph* | 41895 |
| 22 | hand search* | 9708 |
| 23 | manual search* | 6409 |
| 24 | relevant journals | 1534 |
| 25 | search strategy OR search criteria OR systematic search OR study selection OR data extraction | 95820 |
| 26 | search the literature OR searched the literature OR literature search OR literature searched OR literature was searched | 79289 |
| 27 | medline OR pubmed OR cochrane OR embase OR psychlit OR psyclit OR psychinfo OR psycinfo OR CINAHL OR science citation index | 399893 |
| 28 | 3 OR 4 OR 5 OR 6 OR 7 OR 8 OR 9 OR 10 OR 11 OR 12 OR 13 OR 14 OR 15 OR 16 OR 17 OR 18 OR 19 OR 20 OR 21 OR 22 OR 23 OR 24 OR 25 | 847986 |
| 29 | 3 AND 26 | 630 |

Online Supplementary Table 2- Data extraction proforma

| ID and systematic review basic information | Study ID | Extractor initials | | Second extractor initials |
| --- | --- | --- | --- | --- |
|  | Author | | Year | |
|  | Country of first author | | Continent of first author | |
|  | Journal | | | |
|  | Journal code | | | |
|  | Impact Factor (IF) | | | |
|  | Title | | | |
|  | Number of studies included | | | |
|  | Number of participants included | | | |
|  | Number of databases searched | | | |
| Review Content and domains | Objectives | | | |
|  | Review typology | | | |
|  | Meta-analysis performed | | | |
|  | Duplicate review available | | | |
|  | Outcomes | | | |
|  | Reporting domains | | | |
|  | Suggested manuscript grouping | | | |
| PRISMA | Point by point inclusion | | | |
|  | Total score | | | |
|  | Meta-analysis score | | | |
|  | Article Retraction/corrections made | | | |
| AMSTAR | Point by point inclusion | | | |
|  | Total score | | | |
|  | Overall confidence rating | | | |
|  | Article Retraction/corrections made | | | |
| ICENI Themes | Point by point inclusion | | | |
|  | Total score | | | |
|  | Theme scores | | | |

Online Supplementary Table 3- All articles included in the review (baseline demographics only).

| **First Author and year published** | **Journal** | **Title** |
| --- | --- | --- |
| Abecassis 2021 | Neurosurgery Clinical of North America | Craniotomy for Treatment of Chronic Subdural Hematoma |
| Adusumilli 2022 | Journal of NeuroInterventional Surgery (JNIS) | Common data elements reported on middle meningeal artery embolization in chronic subdural hematoma: an interactive systematic review of recent trials |
| Alcala-Cerra 2014 | World Neurosurgery | Efficacy and Safety of Subdural Drains After Burr-Hole Evacuation of Chronic Subdural Hematomas: Systematic Review and Meta-Analysis of Randomized Controlled Trials |
| Almenawar 2014 | Annals of Surgery | Chronic subdural hematoma management: a systematic review and meta-analysis of 34,829 patients |
| Bartek Jr 2019 | Frontiers in Neurology | Clinical Course in Chronic Subdural Hematoma Patients Aged 18-49 Compared to Patients 50 Years and Above: A Multicenter Study and Meta-Analysis |
| Belkhair 2013 | Canadian Journal of Neurological Sciences | One versus double burr holes for treating chronic subdural hematoma meta-analysis |
| Berghauser Pont 2012 | European Journal of Neurology | The role of corticosteroids in the management of chronic subdural hematoma: a systematic review |
| Blaauw 2021 | Journal of Neurotrauma | Prevalence of Cognitive Complaints and Impairment in Patients with Chronic Subdural Hematoma and Recovery after Treatment: A Systematic Review |
| Blaauw 2022 | Acta Neurologica Scandinavia | Pathophysiology of transient neurological deficit in patients with chronic subdural hematoma: A systematic review |
| Chari 2014 | British Journal of Neurosurgery | Recommencement of anticoagulation in chronic subdural haematoma: a systematic review and meta-analysis |
| Chari 2015 | Journal of Neurotrauma | Core Outcomes and Common Data Elements in Chronic Subdural Hematoma: A Systematic Review of the Literature Focusing on Reported Outcomes |
| Chari 2015 | Journal of Neurotrauma | Core Outcomes and Common Data Elements in Chronic Subdural Hematoma: A Systematic Review of the Literature Focusing on Baseline and Peri-Operative Care Data Elements |
| Chari 2015 | Journal of Neurosurgery | Twist-drill craniostomy with hollow screws for evacuation of chronic subdural hematoma |
| Court 2019 | Clinical Neurology and Neurosurgery | Embolization of the Middle meningeal artery in chronic subdural hematoma - A systematic review |
| D'Abbondanza 2014 | Neurological Research | Experimental models of chronic subdural hematoma |
| Dian 2021 | Interventional Neuroradiology | Risk of recurrence of subdural hematoma after EMMA vs surgical drainage - Systematic review and meta-analysis |
| Di Cristofori 2022 | Surgical neurology international | Middle meningeal artery embolization for chronic subdural hematomas. A systematic review of the literature focused on indications, technical aspects, and future possible perspectives |
| Ding 2020 | World Neurosurgery | Subperiosteal versus Subdural Drain After Burr Hole Drainage for Chronic Subdural Hematomas: A Systematic Review and Meta-Analysis |
| Dowlati 2020 | World Neurosurgery | Outcomes of Surgical Evacuation of Chronic Subdural Hematoma in the Aged: Institutional Experience and Systematic Review |
| Edlmann 2020 | Acta Neurochirurgica | Systematic review of current randomised control trials in chronic subdural haematoma and proposal for an international collaborative approach |
| Fahmi 2021 | Clinical Neurology and Neurosurgery | Chronic subdural hematoma-induced parkinsonism: A systematic review |
| Greuter 2020 | Frontiers in Neurology | Type of Drain in Chronic Subdural Hematoma-A Systematic Review and Meta-Analysis |
| Guo 2020 | Frontiers in Neurology | Endoscope-Assisted Surgery vs. Burr-Hole Craniostomy for the Treatment of Chronic Subdural Hematoma: A Systemic Review and Meta-Analysis |
| Haldrup 2020 | Acta Neurochirurgica | Embolization of the middle meningeal artery in patients with chronic subdural hematoma—a systematic review and meta-analysis |
| He 2021 | Neurosurgical Review | Evaluation of the efficacy of atorvastatin in the treatment for chronic subdural hematoma: a meta-analysis |
| Hoffman 2021 | Journal of Clinical Neuroscience | Management of chronic subdural hematoma with the subdural evacuating port system: Systematic review and meta-analysis |
| Holl 2018 | World Neurosurgery | Pathophysiology and Nonsurgical Treatment of Chronic Subdural Hematoma: From Past to Present to Future |
| Holl 2019 | Acta Neurochirurgica | Corticosteroid treatment compared with surgery in chronic subdural hematoma: a systematic review and meta-analysis |
| Ironside 2021 | Journal of NeuroInterventional Surgery (JNIS) | Middle meningeal artery embolization for chronic subdural hematoma: a systematic review and meta-analysis |
| Ivamoto 2016 | World Neurosurgery | Surgical Treatments for Chronic Subdural Hematomas: A Comprehensive Systematic Review |
| Jumah 2020 | Acta Neurochirurgica | Efficacy and safety of middle meningeal artery embolization in the management of refractory or chronic subdural hematomas: a systematic review and meta-analysis |
| Krueger 2021 | World Neurosurgery | Intraparenchymal Hemorrhage After Evacuation of Chronic Subdural Hematoma: A Case Series and Literature Review |
| Kwon 2021 | Complementary Therapies in Clinical Practice | Herbal medicine treatment for patients with chronic subdural hematoma: A systematic review and meta-analysis |
| Liu 2022 | European Review for Medical and Pharmacological Sciences | Local anesthesia with sedation and general anesthesia for the treatment of chronic subdural hematoma: a systematic review and meta-analysis |
| Liu 2014 | Journal of Neurosurgery | Chronic subdural hematoma: a systematic review and meta-analysis of surgical procedures |
| Maliawan 2020 | Open Access Macedonian Journal of Medical Sciences | Hematoma Recurrence in Burr Hole Drainage Compared to Burr Hole Irrigation as Treatment of Chronic Subdural Hematoma: A Systematic Review and Meta-analysis |
| Martinez-Perez 2021 | Neurologia | Endovascular embolisation of the middle meningeal artery to treat chronic subdural haematomas: Effectiveness, safety, and the current controversy. A systematic review |
| Miah 2021 | Neuroradiology | Radiological prognostic factors of chronic subdural hematoma recurrence: a systematic review and meta-analysis |
| Nachiappan 2020 | Neurosurgical Review | Role of prophylactic antiepileptic drugs in chronic subdural hematoma—a systematic review and meta-analysis |
| Nathan 2017 | Neurology | Anticoagulant and antiplatelet use in seniors with chronic subdural hematoma: Systematic review |
| Mang 2021 | World Neurosurgery | Fibrinolytic-Facilitated Chronic Subdural Hematoma Drainage-A Systematic Review |
| Peng 2016 | Cochrane Database of Systematic Reviews | External drains versus no drains after burr‐hole evacuation for the treatment of chronic subdural haematoma in adults |
| Phan 2018 | World Neurosurgery | Resumption of Antithrombotic Agents in Chronic Subdural Hematoma: A Systematic Review and Meta-analysis |
| Poon 2018 | Neurosurgical Review | Association between antithrombotic drug use before chronic subdural haematoma and outcome after drainage: a systematic review and meta-analysis |
| Pranata 2020 | Acta Neurochirurgica | Subperiosteal versus subdural drainage after burr hole evacuation of chronic subdural hematoma: systematic review and meta-analysis |
| Qiu 2017 | Medicine | Effects of atorvastatin on chronic subdural hematoma: A systematic review |
| Sahyouni 2017 | World Neurosurgery | Membranectomy in Chronic Subdural Hematoma: Meta-Analysis |
| Scerrati 2020 | Neurosurgical Focus | To drill or not to drill, that is the question: nonsurgical treatment of chronic subdural hematoma in the elderly. A systematic review |
| Sherrod 2021 | British Journal of Neurosurgery | Preoperative MRI characteristics predict chronic subdural haematoma postoperative recurrence: a meta-analysis |
| Shi 2021 | Frontiers in Neuroscience | Adjuvant Corticosteroids With Surgery for Chronic Subdural Hematoma: A Systematic Review and Meta-Analysis |
| Shlobin 2021 | The Journals of Gerontology: Series A | Surgical Management of Chronic Subdural Hematoma in Older Adults: A Systematic Review |
| Shrestha 2022 | World Neurosurgery | Steroid in Chronic Subdural Hematoma: An Updated Systematic Review and Meta-Analysis Post DEX-CSDH Trial |
| Soleman 2017 | Swiss Medical Weekly | The conservative and pharmacological management of chronic subdural haematoma |
| Srivatsan 2019 | World Neurosurgery | Middle Meningeal Artery Embolization for Chronic Subdural Hematoma: Meta-Analysis and Systematic Review |
| Tang 2022 | Frontiers in Neurology | The Efficacy of Adjuvant Corticosteroids in Surgical Management of Chronic Subdural Hematoma: A Systematic Review and Meta-Analysis |
| Teles 2016 | Brazilian Neurosurgery | Surgical Treatment of Chronic Subdural Hematoma: Systematic Review and Meta-Analysis of the Literature |
| Wang H 2021 | Medicine | The effects of antithrombotic drugs on the recurrence and mortality in patients with chronic subdural hematoma |
| Wang Y 2021 | Frontiers in Aging Neuroscience | Pharmacological Treatment in the Management of Chronic Subdural Hematoma |
| Wang Y 2017 | Journal of Clinical Neuroscience | Influence of antithrombotic agents on the recurrence of chronic subdural hematomas and the quest about the recommencement of antithrombotic agents: A meta-analysis |
| Wan 2019 | World Neurosurgery | Single Versus Double Burr Hole Craniostomy in Surgical Treatment of Chronic Subdural Hematoma: A Meta-Analysis |
| Waqas 2019 | World Neurosurgery | Safety and Effectiveness of Embolization for Chronic Subdural Hematoma: Systematic Review and Case Series |
| Weigel 2003 | Journal of Neurology, Neurosurgery and Psychiatry | Outcome of contemporary surgery for chronic subdural haematoma: evidence based review |
| Wei 2022 | Frontiers in Neurology | Effect of Twist-Drill Craniostomy With Hollow Screws for Evacuation of Chronic Subdural Hematoma: A Meta-Analysis |
| Wu 2018 | World Neurosurgery | Arachnoid Cyst-Associated Chronic Subdural Hematoma: Report of 14 Cases and a Systematic Literature Review |
| Xie 2020 | World Neurosurgery | A Comparison of Subperiosteal or Subgaleal Drainage with Subdural Drainage on the Outcomes of Chronic Subdural Hematoma: A Meta-Analysis |
| Xu 2016 | Neurologia medico-chirurgica | Burr-hole Irrigation with Closed-system Drainage for the Treatment of Chronic Subdural Hematoma: A Meta-analysis |
| Xu 2017 | European Review for Medical and Pharmacological Sciences | Chronic subdural hematoma management: clarifying the definitions of outcome measures to better understand treatment efficacy - a systematic review and meta-analysis |
| Yagnik 2021 | Acta Neurochirurgica | Twist drill craniostomy vs burr hole drainage of chronic subdural hematoma: a systematic review and meta-analysis |
| Zao 2017 | Acta Neurochirurgica | Dexamethasone for chronic subdural haematoma: a systematic review and meta-analysis |
| Yuan 2018 | Medicine | Burr hole drainage and burr hole drainage with irrigation to treat chronic subdural hematoma: A systematic review and meta-analysis |
| Yu 2022 | Frontiers in Pharmacology | Effectiveness Comparisons of Drug Therapy on Chronic Subdural Hematoma Recurrence: A Bayesian Network Meta-Analysis and Systematic Review |
| Zhao 2022 | World Neurosurgery | Efficacy and Safety of Glucocorticoids Versus Placebo as an Adjuvant Treatment to Surgery in Chronic Subdural Hematoma: A Systematic Review and Meta-Analysis of Randomized Controlled Clinical Trials |
| Zhu 2022 | EClinicalMedicine | Factors correlated with the postoperative recurrence of chronic subdural hematoma: An umbrella study of systematic reviews and meta-analyses |

Online Supplementary Table 4- Full-text articles screened and excluded from the review, with reasons.

| **First Author** | **Year published** | **Journal** | **Title** | **Reason for Exclusion** | **Comment** |
| --- | --- | --- | --- | --- | --- |
| Alcala-Cerra, G. | 2014 | Neurocirugia (Asturias, Spain) | [Postoperative bed header position after burr-hole drainage of chronic subdural haematoma: systematic review and meta-analysis of randomised controlled trials] | Not in English | This paper was only available in Spanish |
| Ali, S. | 2012 | The American journal of forensic medicine and pathology | Does hypernatremia cause subdural hematoma in children?: two case reports and a meta-analysis of the literature | Not CSDH | This paper did not define Subdural hematoma as acute or chronic |
| Anker-Moller, T. | 2017 | Seminars in Thrombosis and Hemostasis | Evidence for the Use of Tranexamic Acid in Subarachnoid and Subdural Hemorrhage: A Systematic Review | Not CSDH | This paper did not identify any eligible CSDH studies to include in the review. |
| Bakheet, M.F. | 2014 | Stroke | Effect of addition of clopidogrel to aspirin on subdural hematoma | Conference paper | Conference abstract |
| Bakheet, M.F. | 2015 | International Journal of Stroke | Effect of addition of clopidogrel to aspirin on subdural hematoma: meta-analysis of randomized clinical trials | Not CSDH | This paper did not define Subdural hematoma as acute or chronic |
| Balser, D. | 2013 | Neurological Research | Evolving management of symptomatic chronic subdural hematoma: Experience of a single institution and review of the literature | Literature Review | Systematic review not stated in manuscript |
| Belkhair, S. | 2012 | Canadian Journal of Neurological Sciences | Is double burr hole craniostomy better than single burr hole craniostomy in treating adult patients with chronic subdural hematoma (CSH), in terms of reducing the risk of reoperation for CSH? Systematic review and meta-analysis | Conference paper | Conference abstract |
| Borah, N. | 2015 | Indian Journal of Neurotrauma | Contralateral Extradural Hematoma Formation following Evacuation of Chronic Subdural Hematoma: A Case Report and Review of Literature | Literature review | Systematic review not stated in manuscript |
| Brasiliense, L.B.C. | 2019 | Journal of Neurosurgery | Embolization of middle meningeal artery for chronic subdural hematomas: A systematic review and meta-analysis | Conference paper | Conference abstract |
| Catanese, L. | 2020 | Neurology | Oral factor Xa inhibitors and risk of subdural hematoma: COMPASS trial results and meta-analysis | Not CSDH | This paper grouped ASDH, and CSDH together |
| Catanese, L. | 2019 | Stroke. Conference: American Heart Association/American Stroke Association | Effect of oral factor Xa inhibitors on the risk of subdural hematoma: Compass trial results and systematic review | Conference paper | Conference abstract |
| Catanese, L. | 2021 | European Stroke Journal | Aspirin use and risk of subdural hematoma: Updated meta-analysis of randomized trials | Conference paper | Conference abstract |
| Cheng, L. | 2022 | Frontiers in Neurology | The Impact of Preinjury Use of Antiplatelet Drugs on Outcomes of Traumatic Brain Injury: A Systematic Review and Meta-Analysis | Not CSDH | This paper does not explicitly mention CSDH |
| Cheung, K. | 2019 | Fetal Diagnosis and Therapy | Prenatal Diagnosis, Management, and Outcome of Fetal Subdural Haematoma: A Case Report and Systematic Review | Not CSDH | This paper did not define Subdural hematoma as acute or chronic |
| Connolly, B. | 2013 | Stroke | Vitamin K antagonists versus antiplatelet monotherapy and risk of subdural hematoma: Meta-analysis of randomized clinical trials | Conference paper | Conference abstract |
| Connolly, B. | 2014 | Stroke | Vitamin K antagonists and risk of subdural hematoma: Meta-analysis of randomized clinical trials | Not CSDH | This paper did not define Subdural hematoma as acute or chronic |
| Connolly, B. | 2014 | Stroke | Aspirin therapy and risk of subdural hematoma: Meta-analysis of randomized clinical trials | Not CSDH | This paper did not define Subdural hematoma as acute or chronic |
| Dash, C. | 2017 | Journal of Neurosurgery | Unilateral or bilateral drainage for chronic subdural hematoma | Letter/Correspondence | This paper was a letter to the editor |
| De Amorim, R.L.O. | 2014 | Acta Neurochirurgica | Treatment of traumatic acute posterior fossa subdural hematoma: Report of four cases with systematic review and management algorithm | ASDH only | This paper looked at ASDH only |
| De Beer, M.H | 2017 | Neurologist | Spontaneous Spinal Subdural Hematoma | Not CSDH | This paper did not define Subdural hematoma as acute or chronic |
| Gaonker, V.B. | 2021 | World Neurosurgery | Risk Factors for Progression of Conservatively Managed Acute Traumatic Subdural Hematoma: A Systematic Review and Meta-Analysis | ASDH only | This study examined ASDH |
| Hamou, H.A. | 2022 | Deutsches Arzteblatt International | Chronic Subdural Hematoma-Antithrombotics and Thrombotic Complications | Not in English | This paper was only available in German at the time of review |
| Hart, R.G. | 2021 | Journal of Stroke & Cerebrovascular Diseases | Aspirin Use and Risk of Subdural Hematoma: Updated Meta-Analysis of Randomized Trials | Not CSDH | This paper did not define Subdural hematoma as acute or chronic |
| Hika, B. | 2020 | Clinical Neurosurgery | Failure Rates of Conservative Management of Chronic Subdural Hematoma (CSDH): A Systematic Review | Conference paper | Conference abstract |
| Jang, K.M. | 2020 | Journal of Korean Neurosurgical Society | Arachnoid Plasty to Prevent and Reduce Chronic Subdural Hematoma after Clipping Surgery for Unruptured Intracranial Aneurysm : A Meta-Analysis | Not CSDH | This paper studies iatrogenic CSDH, and as not the primary condition of interest. |
| Joseph, J.R. | 2017 | Journal of Neurosurgery: Pediatrics | Blunt prenatal trauma resulting in fetal epidural or subdural hematoma: Case report and systematic review of the literature | Not CSDH | This paper did not define Subdural hematoma as acute or chronic |
| Kolias, A.G. | 2016 | International Journal of Surgery | Twist-drill craniostomy with hollow screws for evacuation of chronic subdural haematoma | Conference paper | Conference abstract |
| Kolias, A.G. | 2017 | Swiss Medical Weekly | The role of pharmacotherapy in the management of chronic subdural haematoma | Letter/Correspondence | This paper was a letter to the editor |
| Krueger, E. | 2021 | Journal of Neurotrauma | Reperfusion intraparenchymal hemorrhage after evacuation of chronic subdural hematoma: A case series and systematic literature rev | Conference paper | Conference abstract |
| Kwan, M. C. | 2012 | World Neurosurgery | Types of postoperative drainage for chronic subdural hematoma: To do it on the table or under? | Letter/Correspondence | This paper is a journal correspondence |
| Laaidi, A. | 2022 | Annals of Medicine and Surgery | Contralateral epidural hematoma after decompressive surgery: Case report and systematic literature review | ASDH only | This paper looked at ASDH only |
| Lega, B.C. | 2010 | Journal of Neurosurgery | Choosing the best operation for chronic subdural hematoma: a decision analysis | Clinical study | This paper is a clinical article of a surgical decision making tool |
| Lynoe, N. | 2017 | Acta Paediatrica | Insufficient evidence for 'shaken baby syndrome' - a systematic review | Not CSDH | This paper did not define Subdural hematoma as acute or chronic |
| Miller, R. | 2010 | The American journal of forensic medicine and pathology | Overrepresentation of males in traumatic brain injury of infancy and in infants with macrocephaly: further evidence that questions the existence of shaken baby syndrome | Not CSDH | This paper did not define Subdural hematoma as acute or chronic |
| Mongardi, L. | 2020 | Frontiers in Neurology | Low-Dose Acetylsalicylic Acid in Chronic Subdural Hematomas: A Neurosurgeon's Sword of Damocles | Literature review | Systematic review not stated in manuscript |
| Omar, A. | 2022 | World Neurosurgery | Meningiomas Associated with Subdural Hematomas: A Systematic Review of Clinical Features and Outcomes | Not CSDH | This paper focussed on meningiomas as the primary outcome, not CSDH |
| Petralia, C. C. T. | 2020 | World Neurosurgery | Effect of Steroid Therapy on Risk of Subsequent Surgery for Neurologically Stable Chronic Subdural Hemorrhage-Retrospective Cohort Study and Literature Review | Literature review | Systematic review not stated in manuscript |
| Poon M.T.C. | 2016 | British Journal of Neurosurgery | Outcomes associated with antithrombotic drug use in patients undergoing chronic subdural hematoma drainage - A systematic review and meta-analysis | Conference paper | Conference abstract |
| Ratilal, B. O. | 2005 | Cochrane Database of Systematic Reviews | Anticonvulsants for preventing seizures in patients with chronic subdural haematoma | Other review | This was a Cochrane review that did not include any eligible papers |
| Ratilal, B. O. | 2011 | Advances & Technical Standards in Neurosurgery | Prophylactic antibiotics and anticonvulsants in neurosurgery | Not CSDH | This paper was generalised towards all neurosurgical procedures, and not just CSDH. |
| Ratilal, B. O. | 2013 | Cochrane Database of Systematic Reviews | Anticonvulsants for preventing seizures in patients with chronic subdural haematoma | Other review | This was a Cochrane review that did not include any eligible papers |
| Rettenmaier, L. A. | 2017 | Case Reports in Neurological Medicine | Acute, Nontraumatic Spontaneous Spinal Subdural Hematoma: A Case Report and Systematic Review of the Literature | ASDH only | This paper looked at ASDH only |
| Rothrock, R. J. | 2019 | World Neurosurgery | Aneurysmal Subarachnoid Hemorrhage with Spinal Subdural Hematoma: A Case Report and Systematic Review of the Literature | ASDH only | This paper looked at ASDH only |
| Sahyouni, R. | 2017 | World Neurosurgery | Chronic Subdural Hematoma: A Historical and Clinical Perspective | Literature review | Systematic review not stated in manuscript |
| Sahyouni, R. | 2017 | World Neurosurgery | Chronic Subdural Hematoma: A Perspective on Subdural Membranes and Dementia | Literature review | Systematic review not stated in manuscript |
| Schuss, P. | 2013 | Journal of Neurosurgery | Aneurysm-related subarachnoid hemorrhage and acute subdural hematoma: Single-center series and systematic review - Clinical article | ASDH only | This paper looked at ASDH only |
| Scotter, J. | 2015 | Emergency Medicine Journal | Prognosis of patients with bilateral fixed dilated pupils secondary to traumatic extradural or subdural haematoma who undergo surgery: A systematic review and meta-analysis | Not CSDH | This paper did not define Subdural hematoma as acute or chronic |
| Shlobin, N.A. | 2022 | Journals of Gerontology Series A: Biological Sciences & Medical Sciences | Erratum to: A Call for Studies Examining the Management of Chronic Subdural Hematoma in Older Adult Cohorts | Letter/Correspondence | This paper is a journal correspondence |
| Shrestha, D. | 2021 | Journal of the Neurological Sciences | Role of dexamethasone in chronic subdural hemorrhage: A systematic review and meta-analysis | Conference paper | Conference abstract |
| Smith, M.D. | 2012 | International Journal of Surgery | Surgical management of chronic subdural haematoma: One hole or two? | Invited/Expert Review | This paper is an invited expert review |
| Srivatsan, A. | 2019 | World Neurosurgery | In Reply to the Letter to the Editor Regarding "Middle Meningeal Artery Embolization for Chronic Subdural Hematoma: Meta-Analysis and Systematic Review" | Letter/Correspondence | This paper is a journal correspondence |
| Starnoni, D. | 2019 | World Neurosurgery | Letter to the Editor Regarding: "Middle Meningeal Artery Embolization for Chronic Subdural Hematoma: Meta-Analysis and Systematic Review" | Letter/Correspondence | This paper is a letter to the editor |
| Suarez, J.I. | 2017 | Neurology | Thromboembolism prevention after chronic subdural hematoma in the elderly | Letter/Correspondence | This paper is a journal correspondence |
| Suarez, J.I. | 2017 | Neurology | Thromboembolism prevention after chronic subdural hematoma in the elderly | Letter/Correspondence | This paper is a journal commentary |
| Teles, A. | 2012 | Journal of Neurosurgery | Management of chronic subdural hematoma: A brazilian national survey and systematic literature review | Conference paper | Conference abstract |
| Umana, G.E. | 2021 | Journal of Neurological Surgery Part A | A Review of Remote Intracerebral Hemorrhage after Chronic Subdural Hematoma Evacuation | Full-text not available | The full-text for this manuscript was not available at the time of review. |
| Vastani, A. | 2021 | World Neurosurgery | Risk Factor Analysis and Surgical Outcomes of Acute Spontaneous Spinal Subdural Hematoma. An Institutional Experience of Four Cases and Literature Review | ASDH only | This paper looked at ASDH only |
| Won, S.Y. | 2017 | Seizure | A systematic review of epileptic seizures in adults with subdural haematomas | Not CSDH | This paper included both ASDH and CSDH |
| Xu, C.S. | 2015 | Annals of Surgery | Inconsistent data resources weaken the quality of research results | Letter/Correspondence | This paper is a letter to the editor |
| Yan, K, | 2016 | Neurological Research | Endoscopic surgery to chronic subdural hematoma with neovessel septation: technical notes and literature review | Literature review | Systematic review not stated in manuscript |
| Yokota, H. | 2021 | Neuro-Chirurgie | Lumbosacral subdural hematoma associated with cranial subdural hematoma and craniocerebral surgery: Three cases and a systemic literature review | Not CSDH | This paper assessed Lumbosacral subdural hematoma, not CSDH. |
| Yu, J. | 2016 | International Journal of Medical Sciences | Clinical importance of the middle meningeal artery: A review of the literature | Not CSDH | This paper includes other pathologies, and not just CSDH |
| Zaben, M. | 2019 | Child's Nervous System | Subdural haematoma in neonates following forceps-assisted delivery: case series and review of the literature | Not CSDH | This paper did not define Subdural hematoma as acute or chronic |

| **Review content- domains** | **Frequency (%)** |
| --- | --- |
| Anatomy, aetiology, pathophysiology | 6 (8.2) |
| Risk factors | 3 (4.1) |
| Symptoms | 7 (9.6) |
| Diagnosis | 3 (4.1) |
| Surgical management | 32 (43.8) |
| Operative techniques | 27 (37.0) |
| Anaesthetic use | 4 (5.5) |
| Nonsurgical management | 37 (50.7) |
| Complications and recurrence | 63 (86.3) |
| Outcomes | 48 (65.8) |
| Survival | 36 (49.3) |
| Functional outcome | 30 (41.1) |
| Quality of life | 0 (0.0) |

Online Supplementary Table 5- Review domains according to pre-defined themes.

Online Supplementary Table 6. Duplicate reporting details among the 73 included reviews.

| **Similar review published** | **Frequency** |
| --- | --- |
| Yes | 29 (39.7) |
| No | 21 (28.8) |
| **Similar review types** | **Frequency** |
| Middle Meningeal artery (MMA) embolization | 9 (16.7) |
| Corticosteroid use | 7 (13.0) |
| Surgical drain use | 6 (11.1) |
| Surgical management types | 5 (9.3) |
| Nonsurgical management types | 5 (9.3) |
| Age | 4 (7.4) |
| Antithrombotic use and outcome | 3 (5.6) |
| Twist drill craniostomy | 3 (5.6) |
| Irrigation | 3 (5.6) |
| Resumption of antithrombotic medication | 2 (3.7) |
| Number of burr holes | 2 (3.7) |
| Core outcome set | 2 (3.7) |
| Radiological predictors of recurrence | 2 (3.7) |
| Statin use | 2 (3.7) |
| **SD= Standard Deviation, IQR= Inter-quartile range** | |

Online Supplementary Table 7. List of reviews with ‘High’ AMSTAR-2 Rating, and ICENI Theme assessed.

| **Author and year** | **Title** | **ICENI theme(s) and questions** |
| --- | --- | --- |
| Alcala-Cerra 2014 | Efficacy and Safety of Subdural Drains After Burr-Hole Evacuation of Chronic Subdural Hematomas: Systematic Review and Meta-Analysis of Randomized Controlled Trials | Surgical Technique (40) |
| Holl 2019 | Corticosteroid treatment compared with surgery in chronic subdural hematoma: a systematic review and meta-analysis | Natural History (35) |
| Nathan 2017 | Anticoagulant and antiplatelet use in seniors with chronic subdural hematoma: Systematic review | Anticoagulant (13-16) |
| Peng 2016 | External drains versus no drains after burr‐hole evacuation for the treatment of chronic subdural haematoma in adults | Surgical Technique (40) |
| Shrestha 2022 | Steroid in Chronic Subdural Hematoma: An Updated Systematic Review and Meta-Analysis Post DEX-CSDH Trial | Natural History (35) |
| Waqas 2019 | Safety and Effectiveness of Embolization for Chronic Subdural Hematoma: Systematic Review and Case Series | MMA Embolisation (43) |
| Yu 2022 | Effectiveness Comparisons of Drug Therapy on Chronic Subdural Hematoma Recurrence: A Bayesian Network Meta-Analysis and Systematic Review | Natural History (35) |
